# Stage-specific fecal and salivary microbiota signatures for noninvasive detection of colorectal cancer

**DOI:** 10.3389/fmicb.2025.1658693

**Published:** 2025-10-03

**Authors:** Zhenzhen Wu, Zhenzheng Zhu, Chenxi Zhou, Zhenyi Lin, Hujia Yang, Guanjun Jiang, Shuning Ding, Jieru Yu, Leitao Sun

**Affiliations:** ^1^Department of Medical Oncology, The First Affiliated Hospital of Zhejiang Chinese Medical University (Zhejiang Provincial Hospital of Chinese Medicine), Hangzhou, Zhejiang, China; ^2^Longyou Branch of Zhejiang Provincial Hospital of Chinese Medicine, Quzhou, Zhejiang, China; ^3^College of Basic Medical Science, Zhejiang Chinese Medical University, Hangzhou, Zhejiang, China; ^4^Academy of Chinese Medical Science, Zhejiang Chinese Medical University, Hangzhou, Zhejiang, China; ^5^Key Laboratory of Neuropharmacology and Translational Medicine of Zhejiang Province, School of Pharmaceutical Sciences, Zhejiang Chinese Medical University, Hangzhou, Zhejiang, China

**Keywords:** colorectal cancer, fecal microbiota, salivary microbiota, 16S rDNA gene sequencing, microbial co-occurrence networks

## Abstract

**Background:**

Fecal and salivary microbiota dysbiosis play a crucial role in the pathogenesis of colorectal cancer (CRC). We investigated whether the fecal and salivary microbiota were altered during colorectal tumorigenesis and evaluated their diagnostic performance.

**Methods:**

We enrolled 30 metastatic CRC patients, 30 nonmetastatic CRC patients, and 30 healthy controls between October 2023 and September 2024. Fecal and salivary samples were collected for microbial profiling via 16S rDNA sequencing and bioinformatics analysis.

**Results:**

Fecal and salivary microbiota composition differed during CRC progression, with salivary microbiota progressively enriched in the gut. In addition, fecal and salivary microbial co-occurrence networks dynamically altered during CRC progression. The natural connectivity of fecal microbial community networks exhibited decreased stability, whereas salivary microbial community networks showed increased stability as CRC progressed. Finally, specific fecal microbial amplicon sequence variants (ASVs) associated with colorectal carcinogenesis enabled precise stage-specific diagnosis of CRC, outperforming salivary ASVs classifiers.

**Conclusion:**

This study elucidates stage-specific microbial dynamics in CRC, providing novel insights into clinical diagnostic strategies.

## Introduction

Colorectal cancer (CRC) is the second leading cause of cancer-related death in both men and women, with an estimated 1.92 million new CRC cases and 0.90 million CRC-related deaths in 2022, of which approximately 0.24 million deaths occur in China (Bray et al., 2024). Screening for CRC among people aged 50–75 years has substantial net benefits (Patel and Dominitz, 2024). However, only 14% of high-risk population in China undergo CRC screening due to the high cost, invasiveness, and cumbersome preparation required for colonoscopy (Chen et al., 2019). In addition, nonspecific symptoms and absence of clinical biomarkers hinder early CRC detection and intervention. Consequently, up to 60% of patients exhibit local or distant metastasis at the time of diagnosis, correlating with poor prognosis with a 5-year survival rate of less than 20% (Biller and Schrag, 2021). Therefore, a valuable means of predicting the onset of CRC and CRC metastasis is needed urgently.

Although the gut microbiota is not a static entity, it serves as a critical interface dynamically interacting with environmental factors and host health (Zmora et al., 2019), contributing to disease pathogenesis through genetic toxicity, signal transduction, inflammation, immunity, and metabolism (Wong and Yu, 2023). CRC remains one of the most life-threatening malignancies, and unhealthily alterations in fecal microbiota composition in high-risk individuals have been proven to contribute to colorectal carcinogenesis (Cui et al., 2024; Liu et al., 2024; Zepeda-Rivera et al., 2024). Fecal microbiota is highly dynamic, with substantial variation in taxonomic diversity across hosts and health states, reflecting intrahost microbial evolution driven by natural selection. For instance, convergent evidence confirms that specific alterations in fecal microbial composition occur in digestive system diseases, such as in CRC (Coker et al., 2022), gastric cancer (Yu et al., 2024), liver cancer (Rajapakse et al., 2023), and precancerous adenomas (Gao et al., 2023), as well as in other non-neoplastic diseases (Wu et al., 2024), Currently, fecal microbiota has been implicated in the diagnosis of CRC. Coker et al. (2022) established a panel of six fecal bacterial species that distinguished CRC from colorectal adenomas (CRA) with satisfactory diagnostic performance upon validation, indicating the fecal microbial dynamics during CRC progression. The oral cavity acts as the entrance to the gastrointestinal tract and harbors a complex salivary microbiome that is closely linked to the fecal microbiota through oral-to-gut microbiota translocation (Schmidt et al., 2019). Significant alterations in the salivary microbiota of CRC patients highlight its potential as a noninvasive biomarker (Zhang et al., 2020). This positions the readily accessible salivary microbiota as a powerful tool for understanding CRC pathogenesis and distinguishing CRC patients from the healthy population. Recent studies have identified that specific oral bacteria, enriched in the intestinal dysbiotic microbiota of CRC patients, can promote the carcinogenic process and possess significant potential for antitumor therapy (Bergsten et al., 2023; Chen L. et al., 2024; Zepeda-Rivera et al., 2024). However, previous studies have overlooked the critical role of oral-to-gut microbiota translocation during CRC progression. Furthermore, the associations of fecal and salivary microbiota with metastatic CRC remain uninvestigated, along with their potential as diagnostic biomarkers for early metastasis detection.

We hypothesize that stage-specific fecal and salivary microbiota signatures evolve during colorectal carcinogenesis, serving as noninvasive biomarkers for detecting early resectable CRC and monitoring metastasis progression. In addition, given the robust evidence of specific oral bacteria colonizing intestinal dysbiotic microbiota in CRC patients, we hypothesize that oral-to-gut microbiota translocation intensifies throughout colorectal carcinogenesis. Fecal and matching salivary samples were concurrently collected from 30 metastatic CRC patients, 30 nonmetastatic CRC patients, and 30 healthy adult volunteers to characterize the microbial profiles using 16S v3v4 region sequencing approach. We profiled fecal and salivary microbiota and constructed co-occurrence networks to elucidate the ecological dynamics of microbial dysbiosis during CRC progression. Simultaneously, Source Tracker analysis was used to evaluate oral–gut microbial translocation dynamics during CRC progression. Based on bioinformatics analysis, we identified the fecal microbial signatures with acceptable prediction accuracy.

## Results

### Participants

Based on strict inclusion criteria, 30 metastatic CRC patients (M group), 30 nonmetastatic CRC patients (NM group), and 30 healthy volunteers as normal controls (NC group) were included. Clinical features involving sex; age; BMI; comorbidities; location of primary tumor lesion; histological type; and serum CEA, CA-199, and CA-125 level were presented in the Table 1. The differences in demographic characteristics among the three groups were statistically insignificant. As for CRC patients, the specific distribution of their primary tumor location was as follows: NM group: 7 left colon cancer (LCC), 10 right colon cancer (RCC), and 13 rectal cancer (RC) as well as M group: 10 LCC, 7 RCC, and 13 RC. A total of 58 of patients were diagnosed with adenocarcinoma in terms of pathological classification, while only two of the M group patients were diagnosed with neuroendocrine neoplasms, thus showing consistency with the distribution characteristics of CRC histological types. In addition, serum tumor markers exhibited a significant difference between the M group and the NM group.

**Table 1:** Participants.

Clinical features	M (*n* = 30)	NM (*n* = 30)	NC (*n* = 30)	*P* value*
Demographics				ns
Male/female Age yr BMI kg/m^2^ Comorbidities Hypertension Diabetes Tumor location	22/8 62.3000 ± 7.1301 21.9492 ± 2.4995 7 7	18/12 58.6333 ± 11.8015 23.4668 ± 3.6404 12 5	13/17 59.466 ± 4.4079 23.5621 ± 3.1257 10 3 N/A	ns ns
LCC RCC RC	10 7 13	7 10 13		
Tumor type Adenocarcinoma NN Tumor markers	28 2	30 0	N/A N/A	ns *
CEA Normal Elevated CA19-9	14 16	24 6		
Normal Elevated CA-125 Normal Elevated	24 6 21 9	30 0 29 1		

Three-line table showed the clinical features. *Represents *p* < 0.05 (CEA: *p* = 0.014; CA19-9: *p* = 0.023; CA-125: *p* = 0.012). NC, normal controls; NM, non-metastatic CRC; M, metastatic CRC; BMI, body mass index; LCC, left colon cancer; RCC, right colon cancer; RC, rectal cancer; NN, neuroendocrine neoplasm; CEA, carcinoembryonic antigen; CA19-9, carbohydrate antigen 19-9; CA-125, carbohydrate antigen 125; N/A, not applicable; N/I, no information; ns, no significant.

### Fecal microbiota composition differed in CRC patients with distinct disease stages

**Figure 1:**
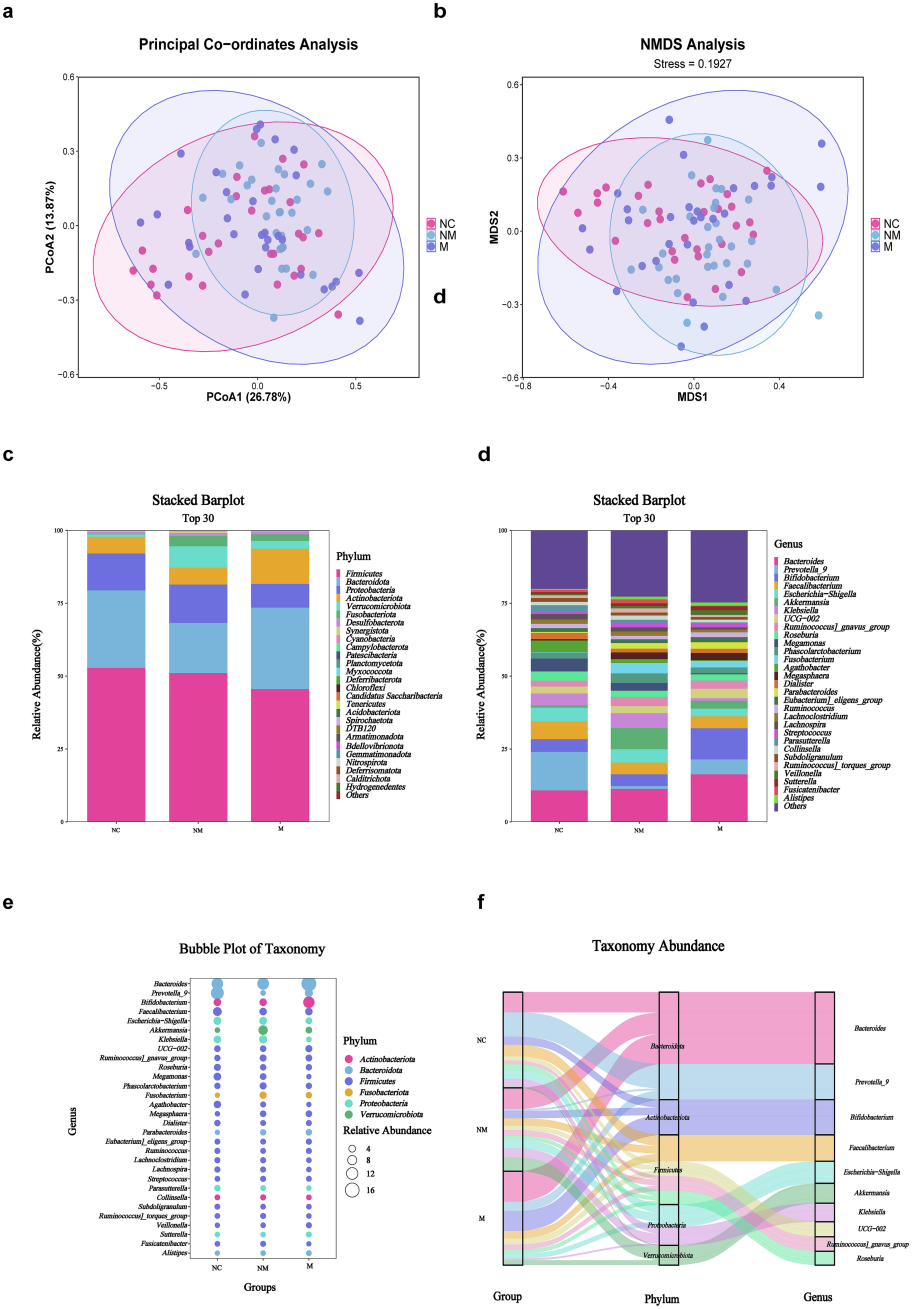
Fecal microbiota composition differed in CRC patients with distinct disease stages. **(a,b)** PCoA **(a)** and NMDS **(b)** showed beta diversity of fecal microbiota across groups with distinct disease stages. *p*-value was calculated by the analysis of similarities (ANOSIM) (weighted Unifrac, R = 0.049, *p* = 0.01). **(c,d)** Stacked bar plot showed mean fecal microbiota proportions at the phylum **(c)** and genus **(d)** levels across groups with distinct disease stages. **(e)** Bubble plot showed fecal microbial genera colored by phylum and sized by relative abundance across groups with distinct disease stages. **(f)** Sankey plot indicated the fecal taxonomic flow with the breadth of the branch at the genus (right side) and corresponding phylum (middle side) levels during the disease progression (left side). NC, normal controls; NM, non-metastatic CRC; M, metastatic CRC.

Intestinal dysbacteriosis is closely associated with colorectal tumorigenesis. Therefore, healthy subjects as well as CRC patients with and without metastasis were enrolled to mirror the specific fecal microbial alterations during CRC progression. Principal coordinates analysis (PCoA) (Figure 1a) and non-metric multidimensional scaling (NMDS) (Figure 1b) analyses revealed significant *β*-diversity shifts (Bray–Curtis, *p* = 0.01) in fecal microbiota, with distinct clustering among the three groups. Moreover, we performed an ASV-based taxonomic analysis at the phylum and genus levels to identify dominant fecal microbiota taxa. At the phylum level, *Firmicutes*, *Bacteroidota*, *Proteobacteria*, *Actinobacteriota*, *Verrucomicrobiota,* and *Fusobacteria* dominated the three groups. In addition, the stacked bar plot of six predominant phyla indicated that the M group had a considerably lower average *Firmicute* composition (45.68%) than the NM (51.18%) and NC group (52.94%), accompanied by the increasing proportion of the *Actinobacteriota* (NC 5.73% vs. NM 5.83% vs. M 12.05%) (Figure 1c). The top 30 genera were plotted in the stacked bar at the genus level, while the rest were merged as others. At the genus level, *Bacteroides* accounted for an increasing proportion along the NC-NM-M sequence (NC 10.92% vs. NM 11.44% vs. M 16.45%) and dominated the NM and M groups. On the contrary, *Prevotella_9* was significantly more abundant in the NC group compared with the CRC group (NC 13.24% vs. NM 0.97% vs. M 5.12%). Furthermore, we observed a progressive decline in the relative abundance of *Escherichia–Shigella* along the NC-NM-M sequence (NC 4.70% vs. NM 4.58% vs. M 2.68%) (Figure 1d). We then shared the top 6 phyla and the top 30 genera in the form of bubble plot and Sankey plot. Bubble plot showed the microbial distribution and changes through the size and location of the bubble among the groups (Figure 1e). The Sankey plot intuitively revealed the branch association between phylum and genus of three groups with different colors (Figure 1f). The average composition of *Bacteroides*, *Prevotella_9*, *Bifidobacterium*, *Faecalibacterium*, *Escherichia-Shigella*, *Akkermansia, Klebsiella*, *UCG-002*, *Ruminococcus_gnavus_group,* and *Roseburia* showed the statistically predominant abundance at the genus level, and *Bacteroidota* showed the largest proportion at the phylum level (Figures 1e,f).

### Specific fecal microbial taxa altered in CRC patients with distinct disease stages

**Figure 2:**
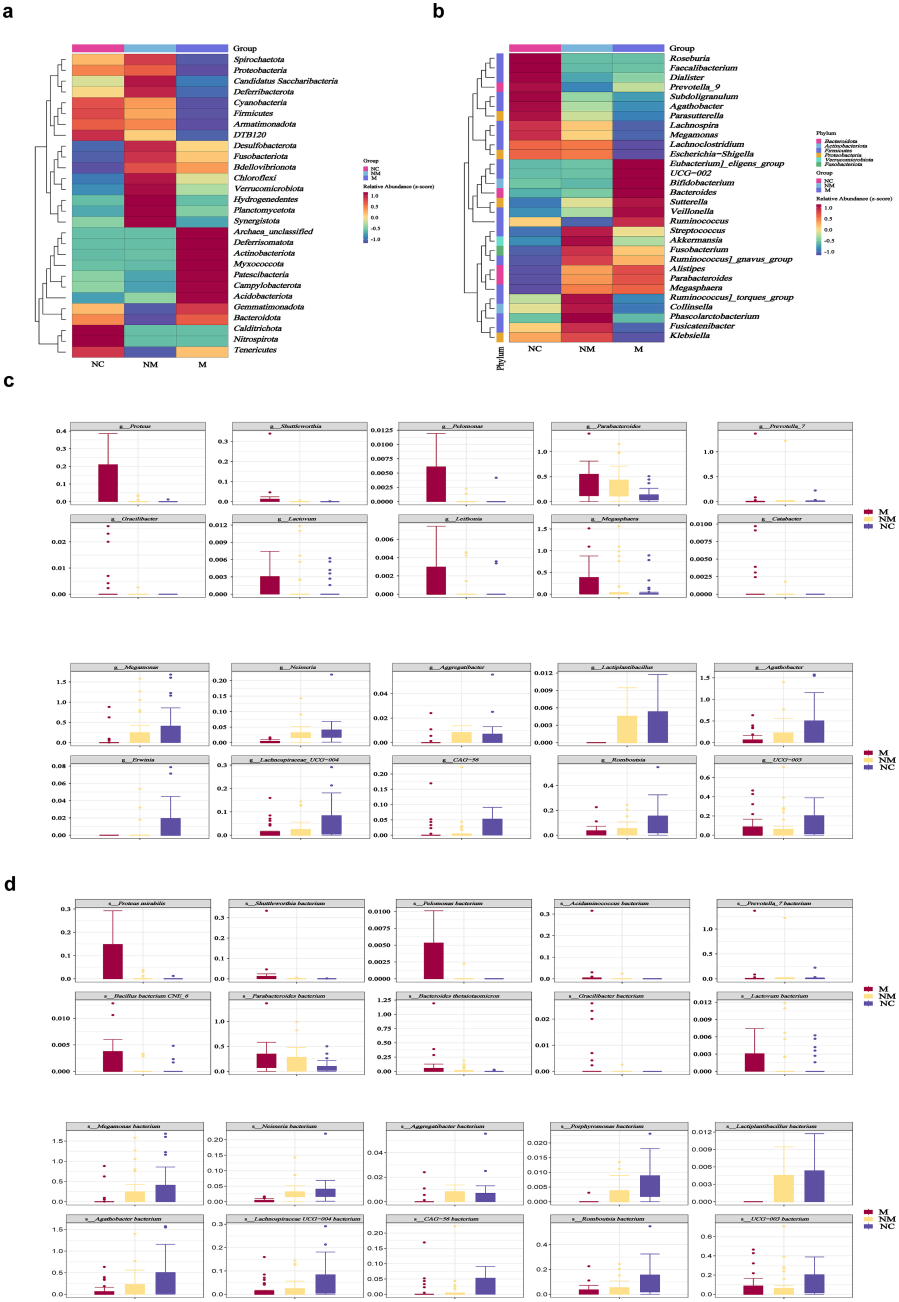
Specific fecal microbial taxa altered in CRC patients with distinct disease stages. **(a,b)** Heatmaps showed fecal microbial phyla **(a)** and genera **(b)** enriched in CRC patients with distinct disease stages. Color gradient indicated relative abundance. **(c,d)** Box plots depicted the specific fecal microbial taxa at the genus **(c)** and species **(d)** levels with progressive relative abundance changes along the NC-NM-M sequence (*p* < 0.05). Species named as unclassified indicated no further classification available. *p*-values were calculated using the Kruskal–Wallis test. The box represented the interquartile range (IQR) between the first and the third quartiles, and the midline represented the median. Whiskers extended to values within 1.5 times IQR. Circles indicated outliers beyond the whiskers. NC, normal controls; NM, non-metastatic CRC; M, metastatic CRC.

Particularly, the heatmap showed difference in microbial distribution at the phylum level (Figure 2a). We observed that the M group featured *Eubacterium eligens group*, *UGG-002*, *Bifidobacterium*, *Bacteroides*, *Sutterella*, *Veillonella, and Ruminococcus,* while the NM group featured *Streptococcus*, *Akkermansia*, *Fusobacterium*, *Ruminococcus gnavus group*, *Ruminococcus torques group*, *Collinsella*, *Phascolarctobacterium*, *Fusicatenibacter*, and *Klebsiella* at the genus level visualized by heatmap (Figure 2b). Box-plots showed the top 10 specific taxa decreased and increased constantly along the NC-NM-M sequence at the genus (Figure 2c) and species (Figure 2d) levels. Specific genera, including *Proteus*, *Shuttleworthia*, *Pelomonas*, *Parabacteroides*, *Leifsonia*, *Megasphaera*, *Catabacter*, *Gracilibacter*, *Lactovum*, and *Prevotella_7* showed increased relative abundance during CRC progression, while others including *Megamonas*, *Neisseria*, *Aggregatibacter*, *Lactiplantibacillus*, *Agathobacter*, *Erwinia*, *Lachnospiraceae UCG−004*, *CAG−56*, *Romboutsia*, and *UCG−003* decreased (Figure 2c). At the species level, *Proteus mirabilis* and *Bacteroides thetaiotaomicron* exhibited an upward trend (Figure 2d).

### Salivary microbiota composition differed in CRC patients with distinct disease stages

**Figure 3:**
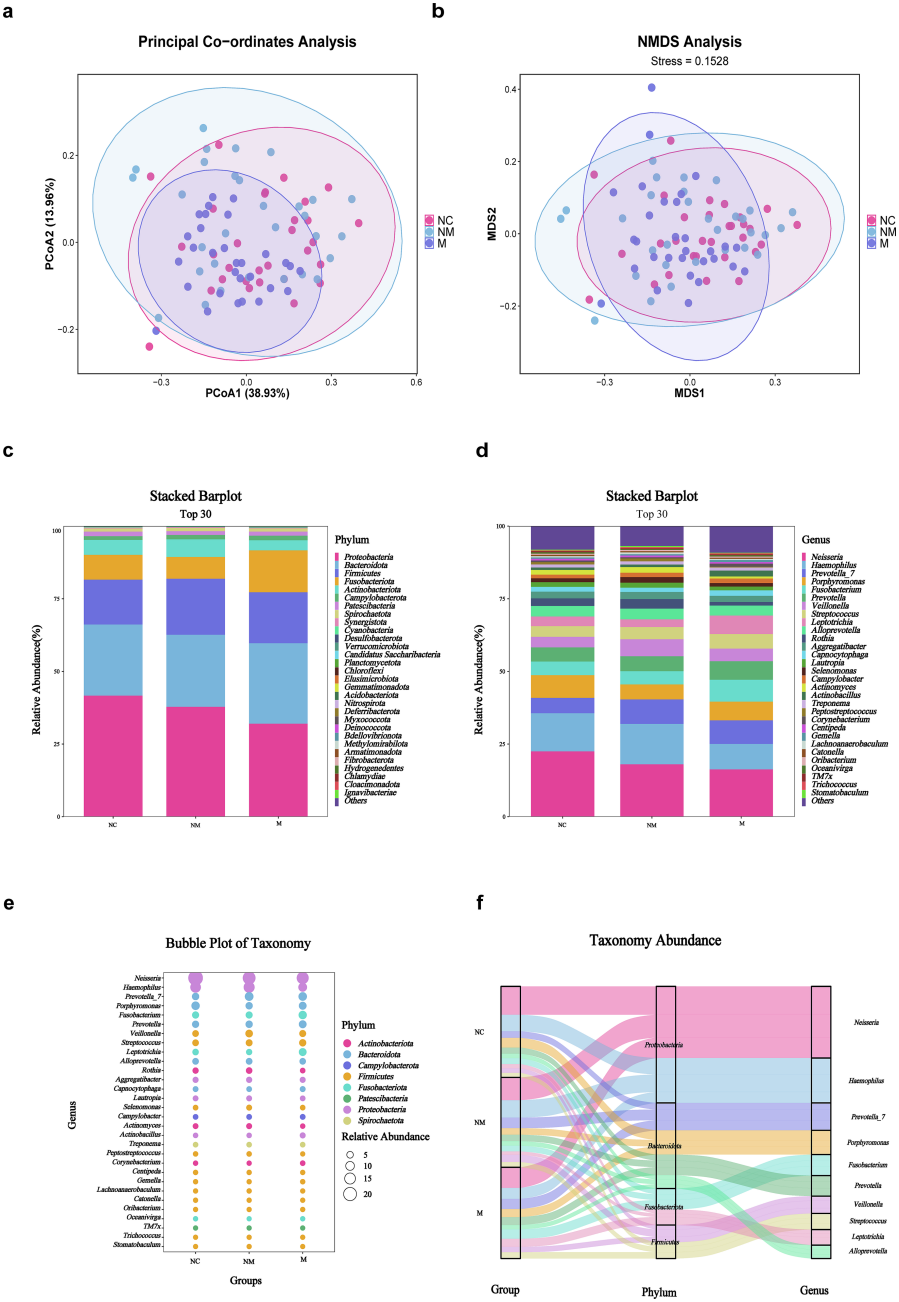
Salivary microbiota composition differed in CRC patients with distinct disease stages. **(a,b)** PCoA **(a)** and NMDS **(b)** showed beta diversity of salivary microbiota across groups with distinct disease stages (weighted Unifrac, R = 0.0797, *p* = 0.001). **(c,d)** Stacked bar plot showed mean salivary microbiota proportions at the phylum **(c)** and genus **(d)** levels across groups with distinct disease stages. **(e)** Bubble plot showed salivary microbial genera across groups with distinct disease stages. **(f)** Sankey plot indicated the salivary taxonomic flow across groups with distinct disease stages. NC, normal controls; NM, non-metastatic CRC; M, metastatic CRC.

A total of 90 salivary samples were available for 16S rDNA sequencing-based taxonomic analysis. Both PCoA (Figure 3a) and NMDS (Figure 3b) analyses demonstrated statistically significant dissimilarities (Bray–Curtis, *p* = 0.001) in microbial community composition among the groups. At the phylum level (Figure 3c), *Proteobacteria*, *Bacteroidota*, *Firmicutes*, *Fusobacteriota*, *Actinobacteriota,* and *Campylobacterota* dominated the three groups. Statistically, *Bacteroidota and Campylobacterota were* enriched in the M group relative to the NM and NC groups, whereas phyla such as *Proteobacteria* showed high abundance in the NC group. Furthermore, *Neisseria*, *Haemophilus*, *Prevotella_7*, *Porphyromonas*, *Fusobacterium*, *Prevotella*, *Veillonella*, *Streptococcus*, *Leptotrichia,* and *Alloprevotella* dominated the three groups at the genus level (Figure 3d). The stacked bar plot depicting the top 30 genera revealed a progressive decline in *Neisseria* abundance during CRC progression, contrasting with increased relative abundances of *Fusobacterium* and *Prevotella* (Figure 3d). To compressively visualize 16S rDNA sequencing data, bubble and Sankey plots were employed to reveal the six most abundant phyla and 30 most abundant genera across the three groups (Figures 3e,f).

### Specific salivary microbial taxa altered in CRC patients with distinct disease stages

**Figure 4:**
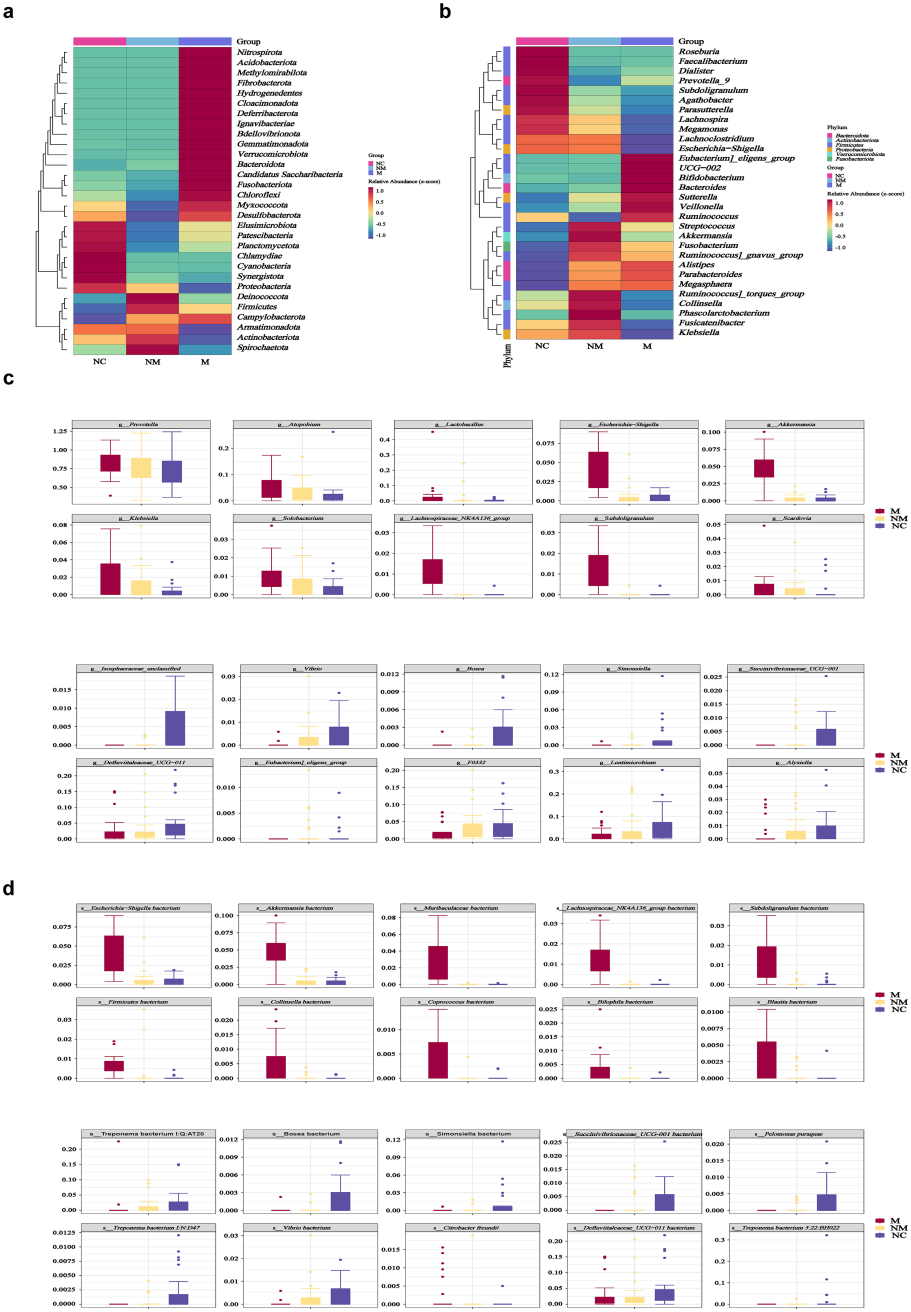
Specific salivary microbial taxa altered in CRC patients with distinct disease stages. **(a,b)** Heatmaps showed salivary microbial phyla **(a)** and genera **(b)** enriched in CRC patients with distinct disease stages. **(c,d)** Box plots depicted the specific salivary microbial taxa at the genus **(c)** and species **(d)** levels with progressive relative abundance changes along the NC-NM-M sequence. NC, normal controls; NM, non-metastatic CRC; M, metastatic CRC.

Particularly, heatmaps illustrated the microbial distribution differences at the phylum (Figure 4a) and genus levels (Figure 4b). Box-plots revealed the top 10 specific taxa that decreased or increased consistently along the NC-NM-M sequence at the genus (Figure 4c) and species (Figure 4d) levels. The relative abundance of specific genera, including *Prevotella*, *Atopobium*, *Lactobacillus*, *Escherichia−Shigella*, *Akkermansia*, *Klebsiella*, *Solobacterium*, *Lachnospiraceae NK4A136 group*, *Subdoligranulum*, and *Scardovia* increased during CRC progression, while others including *Vibrio*, *Bosea*, *Simonsiella*, *Succinivibrionaceae UCG−001*, and *F0332* decreased (Figure 4c).

### Microbiota co-occurrence networks dynamically altered in CRC patients with distinct disease stages

**Figure 5:**
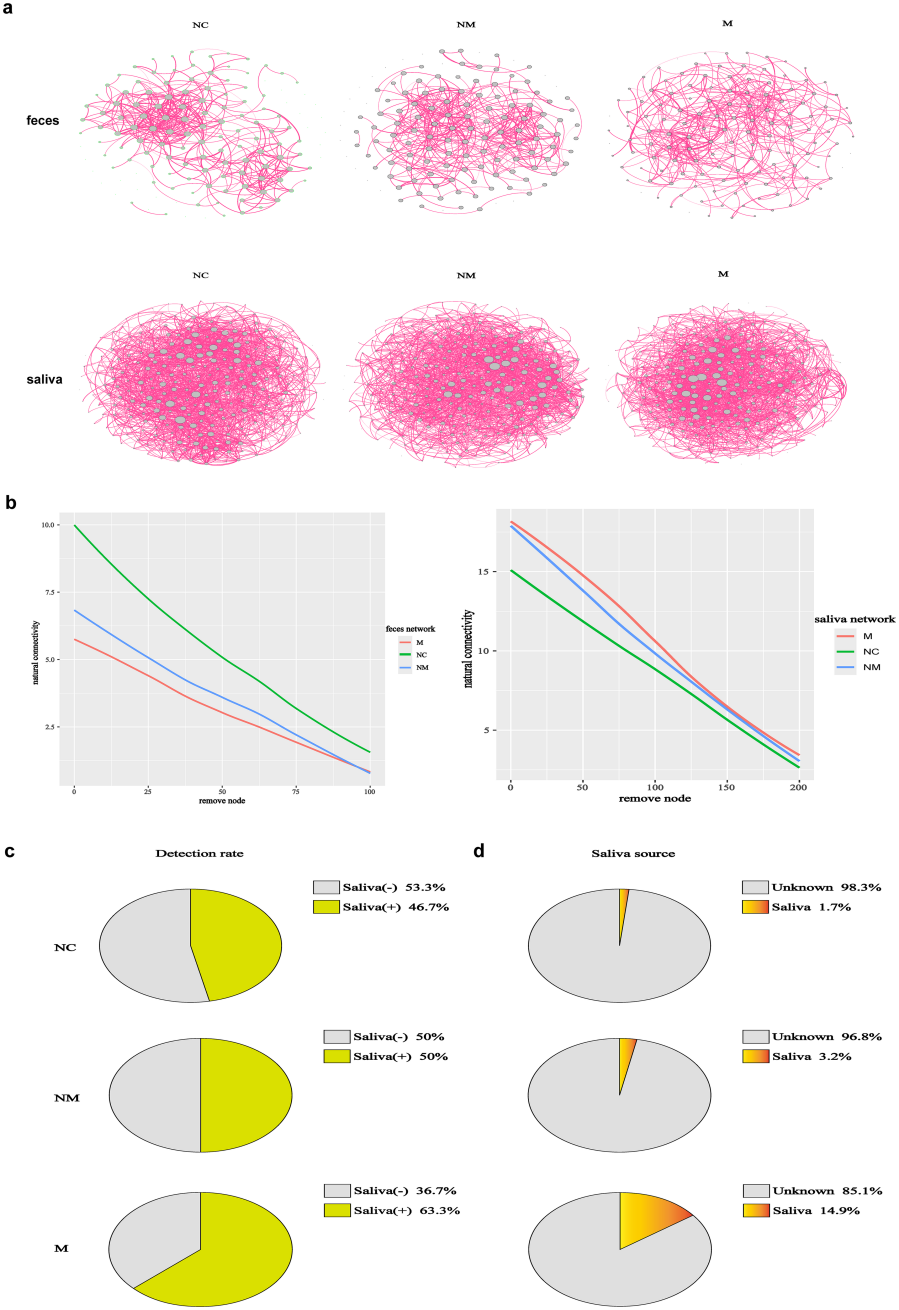
Microbiota co-occurrence networks dynamically altered in CRC patients with distinct disease stages. **(a)** Co-occurrence networks of fecal (*n* = 139) and salivary (*n* = 281) ASVs of CRC patients with distinct disease stages, with edges denoting significant correlations (*p* < 0.05) between co-abundant ASVs. The SparCC algorithm was used to examine statistical significance. **(b)** The network natural connectivity test assessed the robustness changes in two network topologies during node removal. **(c,d)** The source tracker analysis showed both the detection rate and detailed proportion of saliva-derived microbiota in the fecal samples of CRC patients with distinct disease stages. In the pie diagrams of detection rate **(c)**, “Saliva (+)” represented the positive proportion of each group that detected saliva-derived bacteria in their fecal microbiota. “Saliva (−)” represented the negative proportion of each group that did not detect saliva-derived bacteria in their fecal microbiota. In the pie diagrams of the detailed proportion of saliva-derived microbiota in the fecal samples **(d)**, “Saliva” implied the percentage of saliva-derived bacteria in the fecal samples and “unknown” implied the percentage of other bacteria except saliva-derived bacteria (*n* = 30 in the NC, NM, and M groups). NC, normal controls; NM, non-metastatic CRC; M, metastatic CRC.

To assess CRC stage-related changes in gut/oral network complexity, we identified 139 fecal and 281 salivary ASVs (shared by ≥1/3 of samples across groups) and analyzed community structure via SparCC correlation coefficients. We analyzed the interactions among fecal and salivary microorganisms along the NC-NM-M sequence, across 6 networks. Fecal and salivary microbial networks exhibited stage-specific co-occurrence patterns during CRC progression (Figure 5a). In addition, we calculated the natural connectivity to evaluate the resilience in complex networks. Fecal microbial networks demonstrated reduced constancy when the disease progressed, while salivary microbial networks demonstrated increased constancy (Figure 5b). Relative enrichment of oral bacteria in fecal samples has been linked to several digestive system diseases, including Crohn’s disease, ulcerative colitis, irritable bowel syndrome, and even CRC. To investigate whether CRC-induced fecal microbiota changes resulted from oral-to-gut microbiota translocation and intensified with disease progression, we linked salivary and fecal microbiota via Venn diagrams and Source Tracker analysis based on 16S rDNA sequencing. Compared to the NC group, the NM group had more shared ASVs between the salivary and fecal samples as shown in the Venn diagram, suggesting that its salivary and fecal microbiota were more similar (Supplementary Figure S1b). Interestingly, shared ASVs were fewer in the M group than in the NM group (Supplementary Figure S1c). To explore whether oral microbiota colonize the gut during CRC progression, we performed Source Tracker analysis. Source Tracker analysis detected salivary bacteria in the fecal microbiota of 63.3% of M group patients, 50.0% of NM group patients, and 46.7% of NC group patients (Figure 5c), with the highest detection rate observed in the CRC group, particularly the M group. Salivary bacteria constituted 14.9% of the fecal microbiota in the M group, progressively decreasing to 3.2% in the NM group and 1.7% in the NC group (Figure 5d), indicating relative enrichment of salivary bacteria in the gut during CRC progression. Source Tracker details were provided in Supplementary Figures S2a–c. A conceivable circumstance was that oral-to-gut microbiota translocation could drive gut dysbiosis during CRC progression, thereby exacerbating fecal microbial network instability.

### ASVs-based fecal biomarkers achieved precise stage-specific diagnosis of CRC, outperforming salivary ASV classifiers

**Figure 6:**
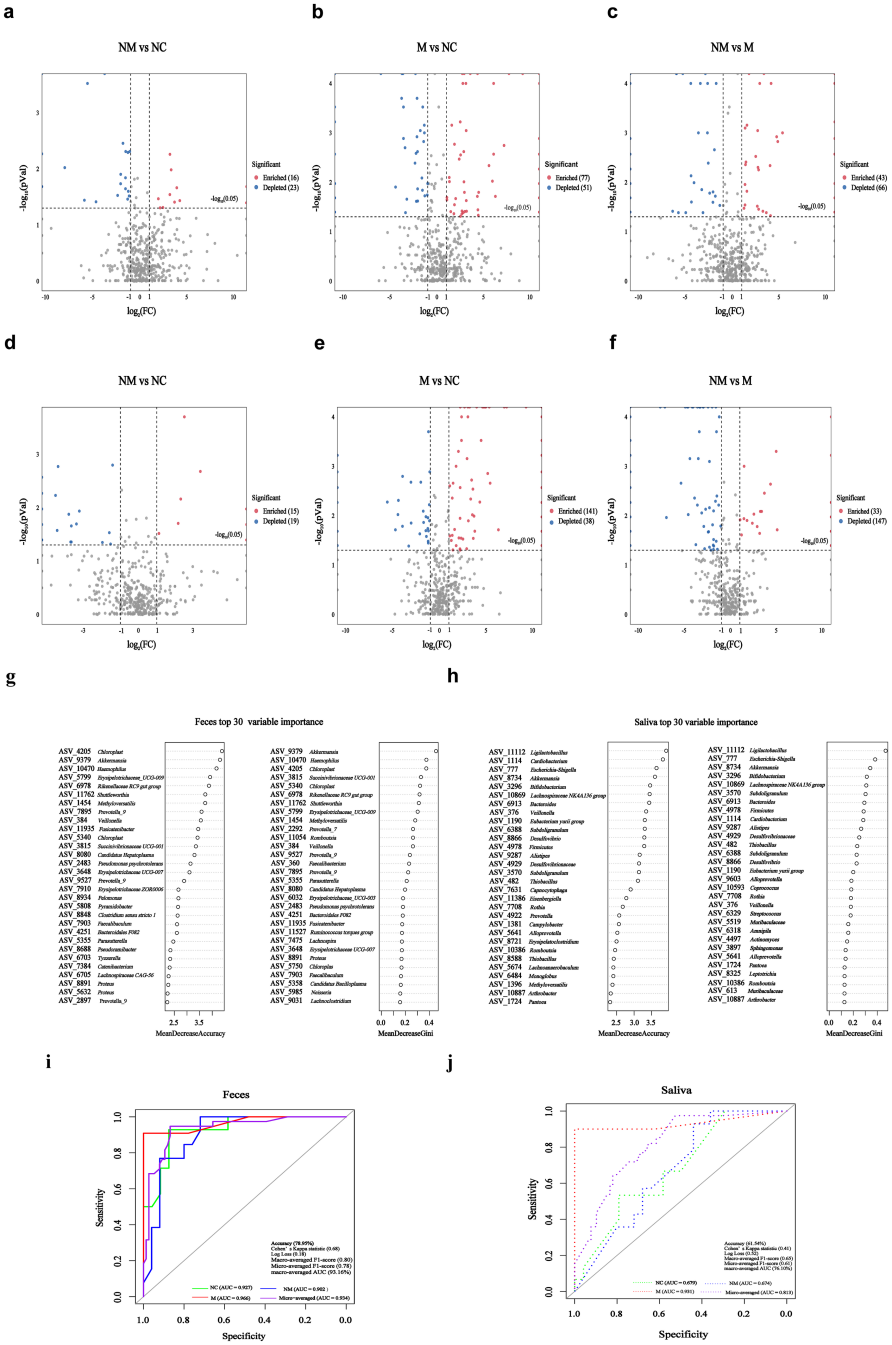
ASVs-based fecal biomarkers achieved precise stage-specific diagnosis of CRC, outperforming salivary ASV classifiers. **(a–f)** Volcano plots revealed significantly altered fecal **(a–c)** and salivary **(d–f)** bacterial species (*p* < 0.05, |log_2_ FC| > 1) in pairwise comparisons among the NM, M, and NC groups. “Enriched:” specific fecal/salivary species were significantly enriched in the front group relative to the rear group; “Depleted:” specific fecal/salivary species were significantly depleted in the front group relative to the rear group; **(g,h)** ASV-based fecal **(g)** and salivary **(h)** biomarkers importance ranking based on random forest scores. **(i,j)** ROC analysis of top eight ASVs-based fecal **(i)** and salivary **(j)** ASVs biomarkers (selected by MeandecreaseGini) for distinguishing among NC, NM, and M groups. NC, normal controls; NM, non-metastatic CRC; M, metastatic CRC; FC, fold change; ASV, amplicon sequence variant; ROC, receiver operating characteristic; AUC, area under the curve.

Differential analysis revealed the number of differential fecal and salivary bacterial species in pairwise group comparisons. Compared to the NC group, the NM group exhibited 39 differential fecal species (16 enriched, 23 depleted) (Figure 6a), while the M group exhibited 128 differential fecal species (77 enriched, 51 depleted) (Figure 6b). Moreover, compared to the M group, the NM group exhibited 109 differential fecal species (43 enriched, 66 depleted) (Figure 6c). Compared to the NC group, the NM group demonstrated 34 differential salivary species (15 enriched, 19 depleted) (Figure 6d), whereas the M group demonstrated 179,179 differential salivary species (141enriched, 38 depleted) (Figure 6e). Furthermore, compared to the M group, the NM group exhibited 180 differential salivary species (33 enriched, 147 depleted) (Figure 6f). Notably, the differences between the M group and the NC group were greater than those between the NM group and the NC group. The results indicated distinct fecal and salivary microbiota differences between CRC patients at different disease stages.

Using the random forest model with five-fold cross-validation, we next ranked fecal (Figure 6g) and salivary (Figure 6h) bacterial ASVs by their diagnostic importance for NC/NM/M three-class discrimination. We observed substantial overlap between the top 30 fecal and salivary ASV biomarkers selected by Mean Decrease Gini score and Mean Decrease Accuracy score. Subsequently, we constructed three-class random forest models using the top 8 fecal and salivary ASV biomarkers (selected by Mean Decrease Gini) to discriminate among NC, NM, and M groups. The fecal model achieved robust discrimination, attaining an overall test accuracy of 78.95%, Cohen’s Kappa statistic of 0.68, Log Loss of 0.18, Macro-averaged F1-score of 0.80, and Micro-averaged F1-score of 0.78. Notably, receiver operating characteristic (ROC) analysis (one-vs-rest) yielded a macro-averaged area under curve (AUC) of 93.16% (NC-specific AUC = 92.71%, NM-specific AUC = 90.15%, M-specific AUC = 96.63%) and a micro-averaged AUC of 93.40% (Figure 6i), confirming balanced performance across classes. The salivary model attained an overall test accuracy of 61.54%, Cohen’s Kappa statistic of 0.41, Log Loss of 0.52, Macro-averaged F1-score of 0.65, and Micro-averaged F1-score of 0.61. ROC analysis (One-vs-Rest) yielded a macro-averaged AUC of 76.15% (NC-specific AUC = 67.92%, NM-specific AUC = 67.43%, M-specific AUC = 93.10%) and a micro-averaged AUC of 81.30% (Figure 6j).

## Discussion

As an aggressive malignancy characterized by insidious onset, rapid metastasis, and high mortality, CRC urgently requires early detection biomarkers to alleviate the disease burden. Recent research has deepened our understanding about the intrinsic links between oral/gut microbiota and digestive tract diseases, particularly CRC (Chen et al., 2022; Baker et al., 2024; Kunath et al., 2024).

Our study identified fecal genera implicated in CRC pathogenesis, including *Bacteroides* (Zafar and Saier, 2021), *Megasphaera* (Chen Q. et al., 2024), *Veillonella* (Ubachs et al., 2021), and *Alistipes* (Gu et al., 2023). In addition, opportunistic pathobionts (e.g., toxigenic *Bacteroides* strains) progressively expanded along the NC-NM-M sequence, whereas commensal probiotics like *Agathobacter* showed significant depletion (Lavelle et al., 2022). *Bacteroides* promoted intestinal barrier permeability in metastatic CRC, thereby shaping a premetastatic niche (Genua et al., 2021). Moreover, Kyoto Encyclopedia of Genes and Genomes (KEGG) analysis identified key metabolic pathways in the M group, notably AA biosynthesis and metabolism (Supplementary Figure S3). This finding was consistent with the prior evidence that AA metabolism accelerated CRC progression (Tian et al., 2020; Tran et al., 2020; Liu et al., 2021). Specifically, western diets depleted beneficial bacteria that produced anti-inflammatory metabolites. In contrast, Mediterranean diet interventions promoted the abundance of *Agathobacter* and enhanced intestinal barrier function via increased production of short chain fatty acids (SCFAs) (Tan et al., 2023; Merra et al., 2020). Intriguingly, these microbial shifts also presented potential therapeutic targets for traditional Chinese medicine. For instance, berberine inhibited pathogenic species such as *Alistipes* and increased the abundance of SCFA-producing bacteria (Yang et al., 2023). We therefore speculated that CRC-associated dysbiosis is both a consequence of deleterious dietary patterns and a malleable “therapeutic interface” that can be modulated through precision interventions. Further research into intervening these microbial imbalances to halt or reverse disease progression holds significant clinical potential.

We tracked CRC stage-dependent co-occurrence network dynamics of fecal and salivary microbiota. Consistent with prior evidence, microbial co-occurrence networks in healthy population exhibited higher stability and complexity than diseased cohorts (Cheng et al., 2022). Structurally integrated networks confer functional robustness and ecosystem resilience (Wagg et al., 2019; Du et al., 2025). Based on the concepts of connectivity and robustness in network theory (Shi et al., 2020), we calculated the natural connectivity of microbial networks. We observed that fecal microbiota networks showed progressive decline in natural connectivity during CRC progression, whereas salivary networks exhibited increasing connectivity. Undoubtedly, an increasing fragility of the fecal microbial network during CRC progression was observed (Figure 5b). However, what we did not expect was that salivary microbial networks exhibited strengthening co-occurrence stability over the same progression (Figure 5b). The results indicated that tumor progression not only altered microbial diversity and composition but also the interaction network stability of both fecal and salivary microbiota. We hypothesized that enhanced robustness in salivary microbiota networks may contribute to fecal network destabilization through oral-to-gut microbiota translocation, potentially influencing host metabolism and tumor promotion. The Source Tracker analysis demonstrated increasing relative abundance of salivary microbiota in feces during CRC progression (Figure 5d), indicating oral-to-gut microbiota translocation-induced dysbiosis. Oral-to-gut microbiota translocation drives colorectal carcinogenesis through pathobiont-mediated barrier disruption and metabolic dysregulation (Park et al., 2021), while simultaneously offering novel theranostic opportunities: stage-specific translocation signatures enable noninvasive liquid biopsy for early metastasis risk stratification, and targeted interventions against key oral pathobionts may halt CRC progression by restoring gut ecological balance.

We elucidated the fecal and salivary microbiota associated with different CRC stages. In addition, we developed a three-classification diagnostic model based on fecal bacterial ASV biomarkers to distinguish among the NC, NM, and M groups, which demonstrated excellent performance. The model achieved AUC values exceeding 90% across all classifications (NC-specific AUC = 92.71%, NM-specific AUC = 90.15%, M-specific AUC = 96.63%, macro-averaged AUC = 93.16%, micro-averaged AUC = 93.40%) (Figure 6i). These results indicated that fecal microbial communities harbored highly informative biomarkers capable of achieving high discrimination accuracy among the three groups, suggesting strong potential for clinical application as a noninvasive auxiliary diagnostic tool. The salivary bacterial ASV-based model showed acceptable but comparatively lower discriminatory power, with a macro-averaged AUC of 76.15% (NC-specific AUC = 67.92%, NM-specific AUC = 67.43%, M-specific AUC = 93.10%) and a micro-averaged AUC of 81.30% (Figure 6j). Notably, the model exhibited relatively low discriminatory power between the NC and NM groups, while performing better in identifying the M group. This suggested that although salivary microbiota may be indicative of advanced or overt pathological states, it held limited value for detecting early or subtle differences. From a clinical inference perspective, the fecal microbiome demonstrated clear advantages over salivary microbiota in this specific classification task, indicating that gut microbial biomarkers may be more suitable for early screening and stratified management of the disease. Although saliva sampling is noninvasive and convenient, its moderate classification performance suggested that it may be better utilized as a supplementary approach or in combination with other biomarkers to enhance overall diagnostic efficacy. Future studies could focus on developing multimodal integrated models that combine microbial information from diverse sources, such as feces and saliva, to build more robust and generalizable clinical prediction tools.

As a preliminary exploratory study, this research identified CRC progression-associated fecal/salivary microbiota biomarkers and established foundational groundwork for future larger scale research. Although the Source Tracker analysis provided insight into potential oral–gut microbiota translocation, the study did not track specific salivary taxa colonizing the gut and lacked direct experimental validation, as it was not originally designed to examine salivary–fecal microbiota associations during CRC progression. In addition, limitation included the relatively small sample size and lack of external validation for predictive models because of the stringent inclusion criteria and the single-center recruitment. We have implemented five-fold cross-validation to enhance model performance and will conduct external validation in multicenter cohorts and prospective studies to assess clinical utility in the future.

## Materials and methods

### Subjects and specimen collection

From March 2024 to January 2025, we recruited 90 participants (30 patients with metastatic CRC, 30 patients with nonmetastatic CRC, and 30 normal control participants) from the First Affiliated Hospital of Zhejiang Chinese Medical University. All metastatic CRC and nonmetastatic CRC subjects were diagnosed by standard colorectal surgery and the control individuals were considered without CRC-related high-risk factors. The inclusion and exclusion criteria for all patients and healthy control participants were the same as described in detail in Supplementary materials. The 16S rDNA gene amplicon of fecal and salivary microbiota and clinical features were obtained. All fecal samples were further analyzed by metagenomic sequencing to acquire fecal microbiota information comprehensively. Fresh fecal and matching salivary samples were collected from 90 participants in sterile plastic pots containing DNA-stabilizing buffer (DNA Shield; LC-Bio Technology Co., Ltd). Participants were strictly refrained from eating, drinking, smoking, or chewing gum for 30 min before saliva collection. The samples were stored at −20 °C for 4 h and subsequently were stored at −80 °C for 24 h for long-term storage until DNA extraction and taxonomic analysis. In addition, clinical data pertaining to age, sex, BMI, and follow-up information were acquired from the same hospital-structured questionnaires. All subjects provided written informed consent. The Clinical Research Ethics Committee of the First Affiliated Hospital of Zhejiang Chinese Medical University approved the study protocol (2024-KLS-015-01).

### DNA extraction, amplification, sequence procession, library construction, and 16S rDNA sequencing analysis

Fecal microbial DNA was extracted using Fecal Genome DNA Extraction Kit (AU46111-96, BioTeke, China) and salivary DNA was extracted using Salivary Genome DNA Extraction Kit (AU46111-96, BioTeke, China), respectively, according to manufacturer’s instructions. The DNA integrity, sizes, and concentrations were generated by Qubit (Invitrogen, United States). The 16S rDNA gene V3–V4 region was amplified using TransStart FastPfu Polymerase with barcode-indexed primers (341F5′-CCTACGGGNGGCWGCAG-3′;805R:5′-GACTACHVGGG TATCTAATCC-3′) in a two-step PCR protocol. The amplification products were then purified by AMPure XT Beads (Beckman Coulter Genomics, Danvers, MA, United States) and were quantified using Qubit (Invitrogen, United States). Quality-filtered reads were obtained and then evaluated using an Agilent 2100 Bioanalyzer (Agilent, United States) and Illumina library quantitative kits (Kapa Biosciences, Woburn, MA, United States), which were further sequenced on the Illumina NovaSeq 6000 (PE250) platform with the default settings for paired-end reads supported by Lc-Bio Technologies Co., Ltd. (Hangzhou, China). Sequencing primer were removed from de-multiplexed raw sequences using cutadapt (v_1.9). Subsequently, paired end reads were merged using FLASH (v_1.2.8). Low-quality reads (quality scores<20), short reads (<100 bp), and reads containing more than 5% “N” records were trimmed by using the sliding-window algorithm method in fqtrim (v_0.94). Quality filtering was performed to obtain high-quality clean tags according to fqtrim. Chimeric sequences were filtered using Vsearch software (v_2.3.4). DADA2 was applied for denoising and generating ASVs.

### Co-abundance ASVs

The ASVs present in one-third of fecal or salivary samples were considered as key ASVs. The correlations among 139 key ASVs of fecal samples and 281 key ASVs of salivary samples were identified by Sparse Correlations for Compositional Data (SparCC) algorithm (*p*-value < 0.05 was considered statistically significant) using R package SpiecEasi (v_1.1.3). Weak correlations (correlation coefficient <0.3 or >−0.3, *p*-value < 0.05) were removed. After filtering, Gephi (v_0.9.2) was used for the visualization of significant co-occurrence interactions.

### Natural connectivity

Co-abundance ASVs in the corresponding networks, represented as the N nodes, were removed one by one. The natural connectivity statistics (y axis) reflect to invulnerability were calculated when each node (x axis) was removed. The natural connectivity value ( $\bar{\lambda )}$ was calculated by R package igraph (v_4.4.3), it is related to a function $\lambda_{i}$ , which represents the eigenvalues of the network adjacency matrix and can be used to calculate the decrease in redundancy of alternative pathways as nodes are removed, i.e.:


$$\bar{\lambda }=\text{In}(\frac{1}{N}\sum_{i=1}^{N}e^{\lambda_{i}})$$

Where *i* represented the *i*th co-abundance ASVs.

### Random forest classifier

The machine learning analysis utilized the normalized relative abundance of fecal and salivary ASVs. We implemented a three-class Random Forest Classifier, with model performance rigorously quantified through five-fold cross-validation. Evaluation metrics included: classification accuracy, Cohen’s Kappa statistic, logarithmic loss (Log Loss), macro-averaged F1-score, and micro-averaged F1-score. Feature selection and model optimization were performed using R packages randomForest (v_4.4.3) and caret (v_4.4.3) packages. Only the top eight ASVs ranked by meandecreaseGini impurity were retained as predictive features. Diagnostic efficacy was further evaluated using ROC analysis (one-vs-rest) implemented in the R package pROC (v_4.4.3). Model performance was assessed by macro-averaged area under the curve (macro-AUC) and micro-averaged area under the curve (micro-AUC).

### Statistical analysis

Statistical analyses were carried out in R version 4.4.2 to identify microbial differences among groups. Wilcoxon rank-sum test was performed in the pairwise comparisons, while Kruskal–Wallis test was used for multiple group comparisons. Benjamini–Hochberg false-discovery rate (FDR) was used to control the false positive rate in multiple comparisons and ensure the credibility of the statistical results. Analysis of variance (ANOVA) was used to compare age and BMI among M, NM, and NC groups; Pearson’s chi-squared test was used to compare gender distribution differences among the three groups. Fisher’s exact test was used to compare the distribution difference of tumor location, tumor type, and tumor markers between the M group and the NM group. *p* < 0.05 was considered significant.

## Data Availability

The datasets presented in this study are publicly available. This data can be found here: https://ngdc.cncb.ac.cn/gsa, accession number CRA025003.
