# Supplementary material for: Stage-specific fecal and salivary microbiota signatures for noninvasive detection of colorectal cancer
Source: Front Microbiol. 2025 Oct 3;16:1658693. doi: 10.3389/fmicb.2025.1658693 (PMC12531261; doi:10.3389/fmicb.2025.1658693)
Supplement: Supplementary file 1 [file Supplementary_file_1.docx]

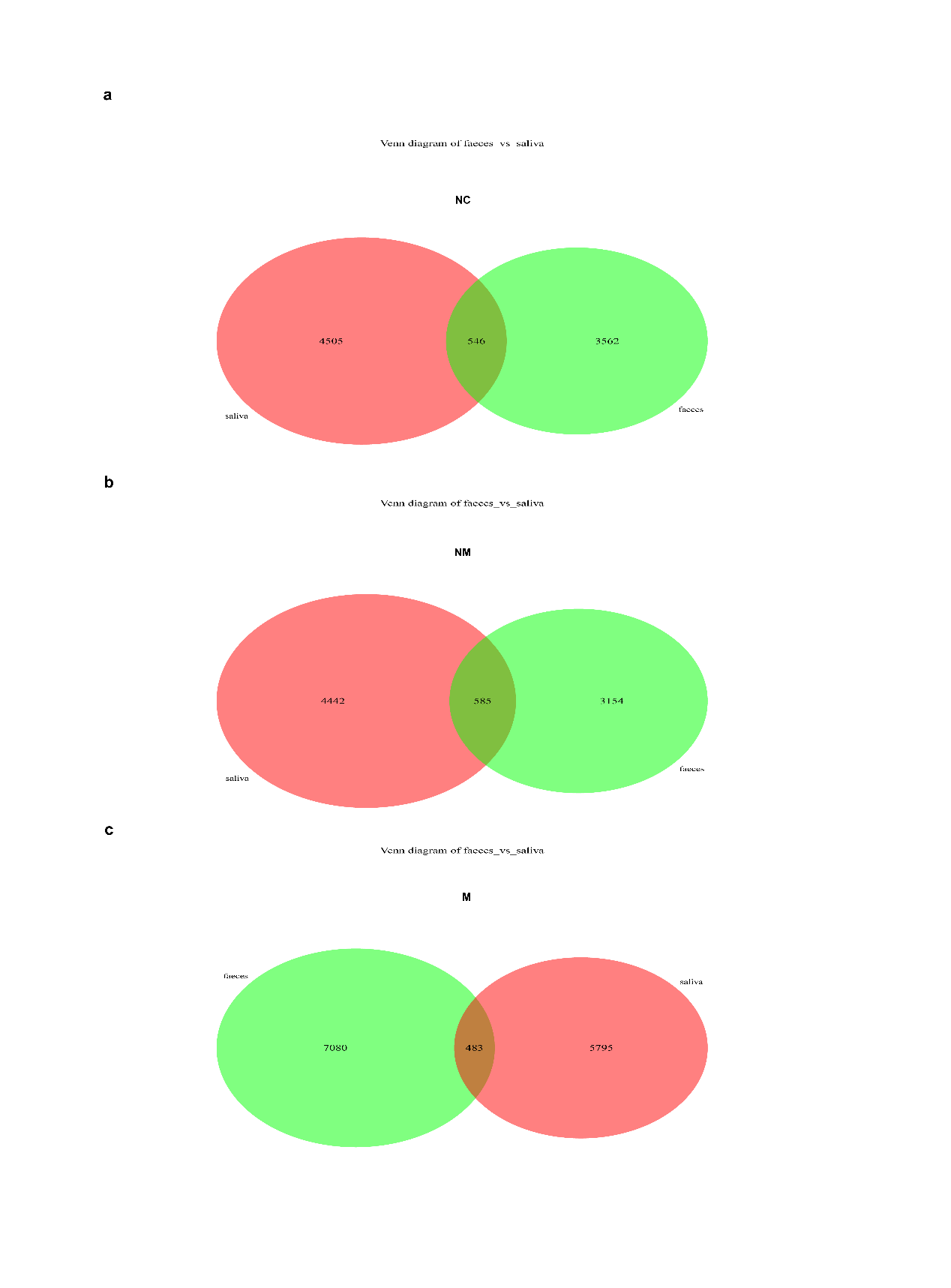


**Fig S1. Shared ASVs between the salivary and fecal samples along CRC stages.**

(a) The shared ASVs of NC group. (b) The shared ASVs of NM group. (c) The shared ASVs of NC group.


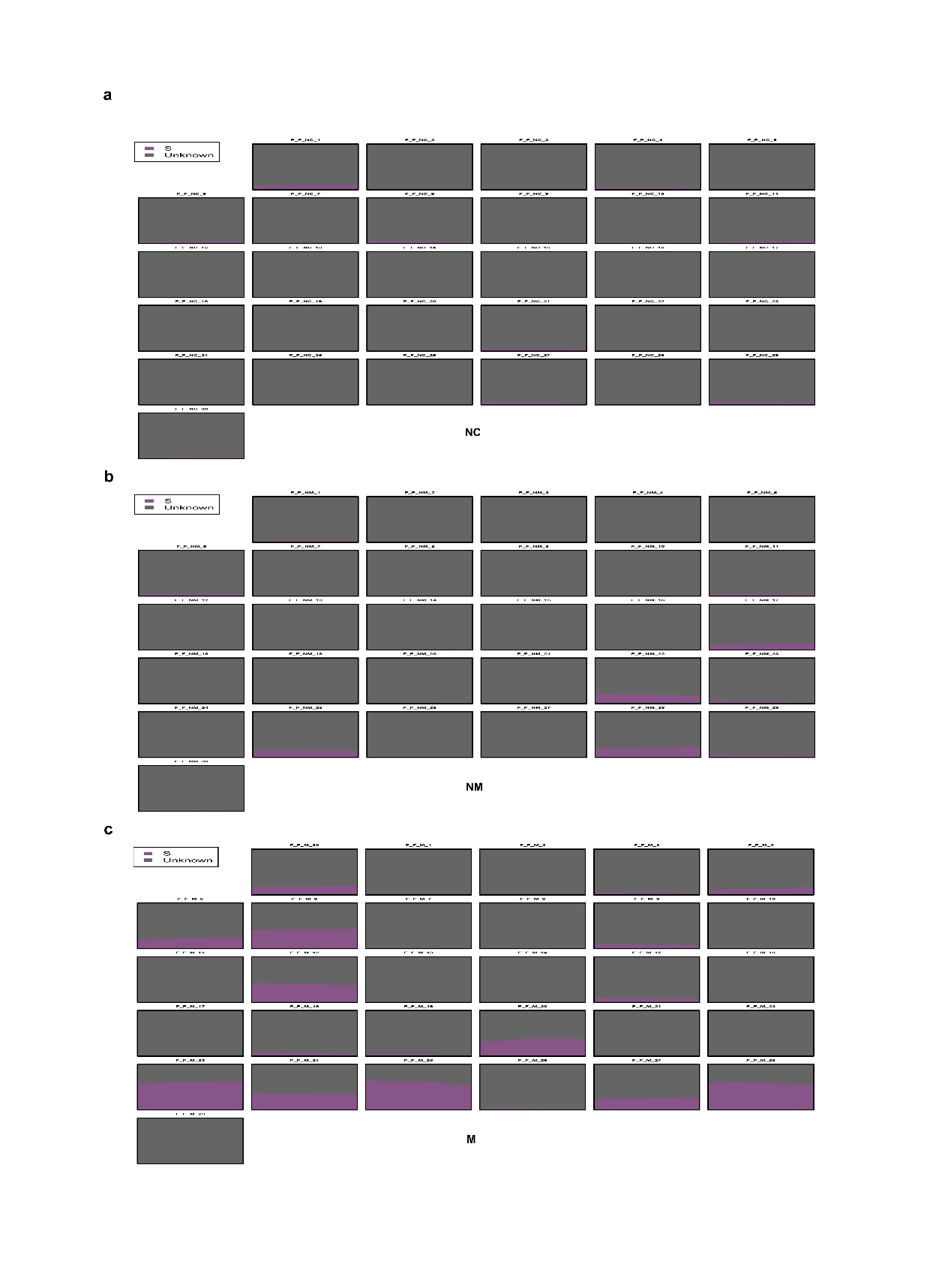


**Fig S2. Detection rate and detailed proportion of saliva-derived microbiota in the fecal samples.**

(a) The result of source tracker analysis of NC group. (b) The result of source tracker analysis of NM group. (c) The result of source tracker analysis of M group.


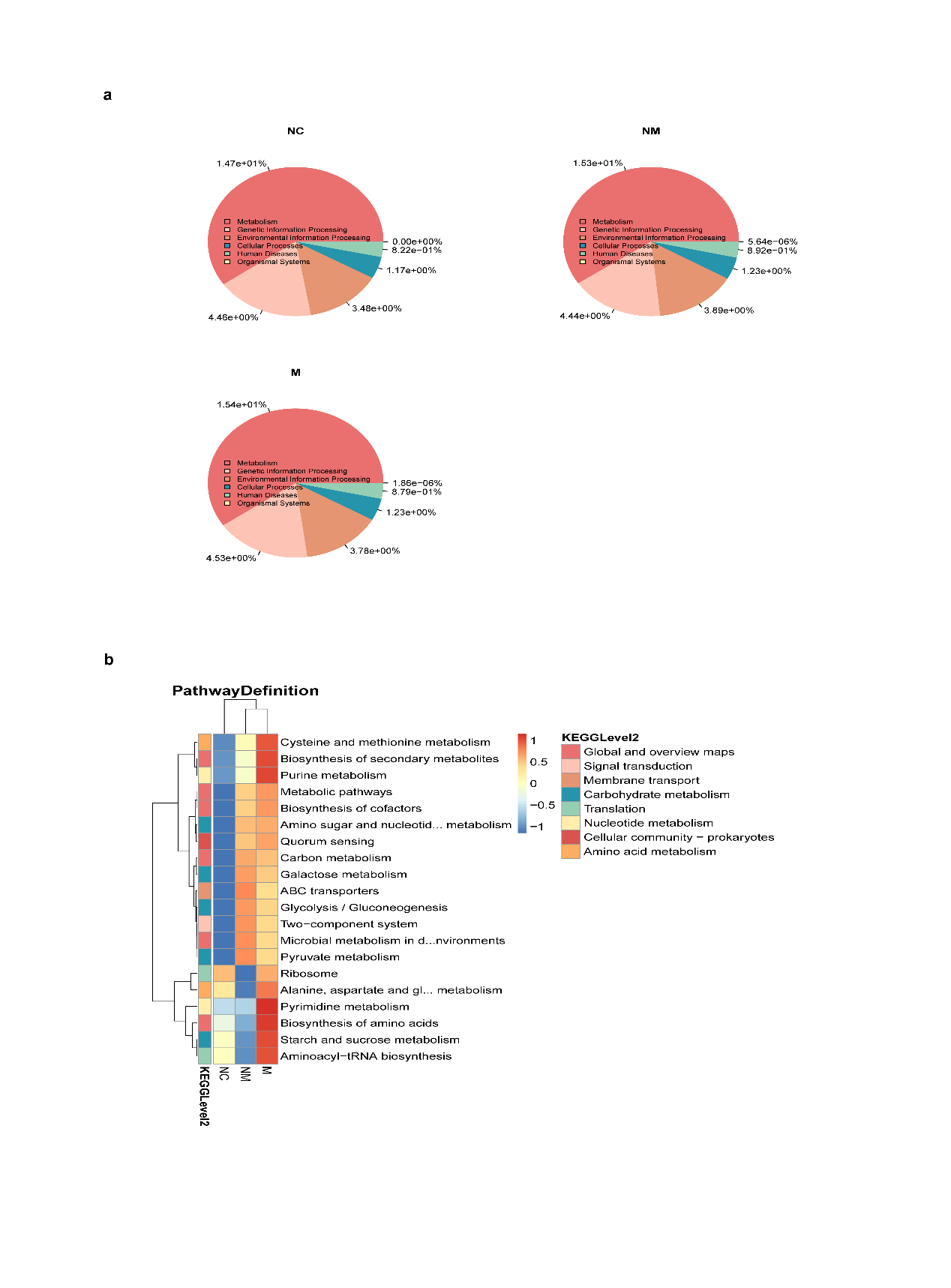
**Fig S3. KEGG pathway enrichment analysis of fecal samples.**

(a) The pie diagram showed the percentage of different pathways in NC, NM and M groups. (b) The heat map showed the enrichment pathways in NC, NM and M groups.

**Inclusion criteria for the metastatic CRC group:**

(a) confirmed CRC with ≥1 metastatic site pathologically;

(b) expected survival period ≥3 months;

(c) no family history of CRC;

(d) aged 18 -75 years;

(e) no diarrhea, vomiting, nausea or other significant gastrointestinal discomfort in the month before enrollment;

(f) no antibiotic or probiotic use in the 4 weeks prior to feces and saliva sample collection.

**Inclusion criteria for the non-metastatic CRC group:**

(a) pathologically confirmed stage II-III CRC;

(b) other criteria were as previously described.

**Inclusion criteria for the healthy individuals:**

(a) no clinical or histopathological CRC diagnosis and asymptomatic;

(b) other criteria were as previously described.

**Exclusion criteria:**

(a) pregnant or breastfeeding women;

(b) patients with inflammation, infectious diseases, or other autoimmune diseases;

(c) individuals with fecal diversion via fistula;

(d) non-compliant individuals.

**Diagnostic criteria for both metastatic and non-metastatic CRC groups**: no other malignancy history except pathologically and surgically confirmed CRC. Case classification and grouping based on the 8th edition of the AJCC TNM classification.
